# Quadriceps combined with hip abductor strengthening versus quadriceps strengthening in treating knee osteoarthritis: a study protocol for a randomized controlled trial

**DOI:** 10.1186/s12891-018-2041-7

**Published:** 2018-05-15

**Authors:** Yujie Xie, Chi Zhang, Wei Jiang, Juan Huang, Lili Xu, Guoyin Pang, Haiyan Tang, Ruyan Chen, Jihua Yu, Shengmin Guo, Fangyuan Xu, Jianxiong Wang

**Affiliations:** Rehabilitation Medicine Department of the Affiliated Hospital of Southwest Medical University, Luzhou, Sichuan People’s Republic of China

**Keywords:** Hip abductor, Quadriceps, Strengthening, Knee osteoarthritis

## Abstract

**Background:**

Lower limb **s**trengthening, especially the quadriceps training, is of much necessity for patients with knee osteoarthritis (KOA). Previous studies suggest that strengthening of the hip muscles, especially the hip abductor, can potentially relieve the KOA-associated symptoms. Nevertheless, the effects of quadriceps combined with hip abductor strengthening remain unclear. Therefore, the current randomized controlled trial is designed aiming to observe whether quadriceps in combination with hip abductor strengthening can better improve the function and reduce pain in KOA patients than quadriceps training alone.

**Methods:**

A total of 80 subjects with symptomatic KOA will be recruited from the communities and hospital outpatient, and will be randomly assigned to the experiment group (Quadriceps-plus-hip-abductor-strengthening) or the control group (Quadriceps-strengthening). Specifically, participants in the experiment group will complete 4 exercises to train the quadriceps and hip abductor twice a day for 6 weeks at home, while those in the control group will only perform 2 exercises to strengthen the quadriceps. Besides, all patients will also receive usual care management, including health education and physical agent therapy when necessary. Knee pain will be measured using the Visual Analogue Scale (VAS) at baseline, in every week during the course of treatment, as well as 8 and 12 weeks after randomization. Furthermore, knee function will be measured using the Western Ontario and McMaster Universities Osteoarthritis Index (WOMAC) scale, and the quality of life will be measured using the MOS Item Short-form Health Survey (SF-36). In this study, several simple tests will be applied to assess the objective function. All the assessments except for VAS will be carried out at baseline, and in the 6th, 8th and 12th weeks respectively.

**Discussion:**

Our findings will provide more evidence for the effects of hip abductor strengthening on relieving pain and improving function in KOA patients. Hip abductor strengthening can be added into the muscle training program for KOA patients as a supplementary content if it is proved to be effective.

**Trial registration:**

The current study has been registered with the Chinese Clinical Trials Registry (the registration number is ChiCTR-IOC-15007590, 3rd December, 2015).

## Background

Knee osteoarthritis (KOA) is one of the most common chronic progressive, degenerative and debilitating diseases among the elderly [1]. KOA patients mostly suffer from progressive stiffness and knee pain. Gradually, they have some difficulties in performing daily activities, such as walking, squatting, climbing and doing housework, as the disease progresses [2]. Moreover, the KOA-induced pain and disability will impair their independence and remarkably reduce their quality of life, thus posing a heavy burden on both the families and the society [3]. Generally, the high prevalence, disability rate and medical expenses of KOA have gradually attracted extensive attention from the public [4].

KOA is a chronic progressive and debilitating disease; however, effective fundamental treatments are lacking at present. The existing clinical treatment strategies for KOA aim to relieve pain and symptoms, as well as to delay disease progression, including pharmacological strategies (such as analgesics, non-steroidal anti-inflammatory drugs, corticosteroids and cartilage protecting agents), non-pharmacological strategies (physiotherapies like ultrasonic treatment, acupuncture, patient education, weight loss, exercises and sports support) [5, 6], and surgical strategies (such as replacement of knee for end-stage KOA) [7]. Certainly, these treatments are helpful; nonetheless, some of them may be associated with many potential side effects and are uneconomical. In addition, some of them may be inconvenient for patients requiring frequent hospital visits, which will inevitably add to additional costs. It is noteworthy that, the latest guidelines for KOA have paid ample attention to some non-pharmacological strategies in disease management [5, 6, 8], particularly exercise rehabilitation and sports support.

A variety of exercises have been proposed in literature to treat KOA, including aerobic exercises such as cycling or walking, as well as some targeted exercises like strengthening of particular muscles, and some exercises targeting flexibility. To the best of our knowledge, studies investigating the effects of muscle strengthening on KOA patients mostly focus on the quadriceps [9]. Meanwhile, studies also find that quadriceps strengthening contributes to relieving pain, improving body function as well as quality of life of patients, and delaying disease progression. Furthermore, it is also characteristic of its safety and economical efficiency, which are extremely important for patients. Therefore, such treatment is unanimously recommended in various guidelines.

However, the lower limb represents a whole chain of motion, and changes in the biomechanical environment of the hip, such as in the muscle strength around the hip, may also affect the knee joint. As is discovered in recent studies, some knee diseases, such as patellofemoral pain syndrome [10, 11], iliotibial band syndrome [12], and noncontact anterior cruciate ligament injuries, are related to the strength of muscles around the hip. Notably, the mechanical and kinematic parameters of hip movement in KOA patients are quite different from those in the normal control group [13–15]. In the meantime, some latest studies also propose that, KOA patients are suffering from decreasing hip abductor muscle strength [16, 17]. Specifically, results from a recent cohort study suggest that, people having a smaller external hip adduction moment (in other words, a weaker hip abductor) are undergoing a faster KOA progression [18]. Moreover, a few studies find that, hip abductor strengthening may potentially alleviate pain and improve the overall functions of these patients [19–21]. However, it can hardly decrease the knee load, as is measured by the knee adduction moment [20]. Nonetheless, the efficacy of hip abductor strengthening has not been fully confirmed by original studies yet, which has prevented the routine prescription of such exercises for KOA patients.

Given that hip abductor strength may potentially affect knee load, it is supposed in the current study that quadriceps combined with hip abductor strengthening may be superior to quadriceps strengthening alone for KOA patients. Thus, the current study is thereby designed on this basis, aiming to determine whether patients receiving quadriceps combined with hip abductor strengthening can attain more functional improvement, more pain relief and higher quality of life than those undergoing quadriceps strengthening alone.

## Methods

### Design

**Fig. 1:**
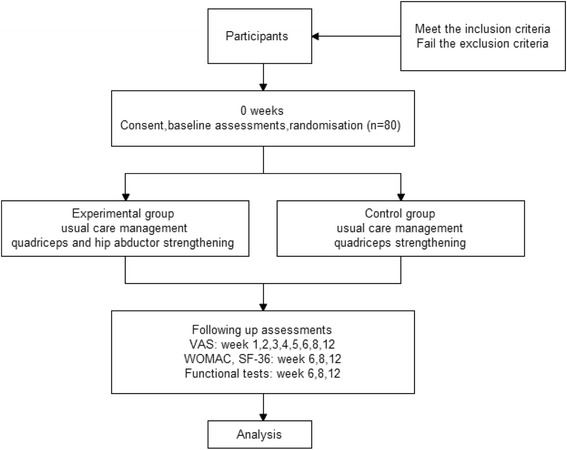
Trial protocol

The current study is a single-blind randomized controlled trial, in which patients with KOA will be randomly divided into quadriceps-plus-hip abductor-strengthening group and quadriceps-strengthening group (Fig.1). Importantly, physicians carrying out the assessments will not be informed of the group situation.

### Study population and allocation

A total of 80 KOA patients will be recruited from the outpatient of the Affiliated Hospital of Southwest Medical University, as well as from some communities in the city by means of advertisements. The inclusion criteria are as follows: those with osteoarthritis in at least one knee according to the American College of Rheumatology Classification Criteria established in 1995 [22]; those aged over 50 years; those with the self-reported average knee pain > 2.0 and < 7.0 measured by the Visual Analogue Scale (VAS) for most days in the month; and those with the Kellgren-Lawrence grade (K-L grade) for KOA of II-IV. KOA will be confirmed through physical examinations and radiography. For participants with bilateral KOA, the more seriously affected side (as identified by K-L grade of KOA and the pain intensity) would be selected as the affected leg.

In the meantime, the exclusion criteria are shown as follows: patients with the age of ≥70 years; those undergoing hip or knee muscle training in the past 4 weeks; those with obvious knee varus or valgus deformity; those who have taken oral corticosteroid within the last 4 weeks or intraarticular corticosteroid injection within the last 3 months; those with systemic arthritic conditions; those who had undergone tibial osteotomy, hip or knee joint replacement, or other surgeries of knee or hip joint; and those with any other neurological, muscular or joint diseases that may affect the lower limb function.

Patients will be recruited in strict accordance with the inclusion and exclusion criteria, and will be subsequently assigned randomly into the Quadriceps-plus-hip-abductor-strengthening group (experiment group) and the Quadriceps-strengthening group (control group) at the ratio of 1:1. Specifically, the randomization sequence will obtained by an independent researcher not taking part in the treatment or assessments using the random number function in Excel. Allocation will be hiden in serial number, with opaque envelopes placing in the central location. In addition, another researcher participating in the study will then open the envelopes in sequence after patient recruitment and baseline assessment.

### Interventions

All participants will receive routine care management for KOA, including health education and physical agent therapy when necessary.

As some guidelines suggested, all KOA participants in both groups will undergo the Quadriceps-strengthening training composed of 2 exercises designed to strengthen the quadriceps. The first exercise is straight leg raise, to be specific, patients lie in supine position, and keep their legs straight with a resistance band placed just proximal to the ankle of the affected limb. Afterwards, they raise the affected leg to the heel 25-30 cm away from the bed, stay in this position for 5–10 s based on their own abilities, and then slowly lay down. The second exercise is multi-angle static exercise. Briefly, patients sit on their seats with a resistance band positioned proximal to the ankle, and contract their quadriceps isometrically for 5–10 s when their knees are flexed at the angles of 0°, 30°, 60°, 90° and 120°, respectively.

Correspondingly, the experiment group will receive Quadriceps-plus-hip-abductor-strengthening training, which is constituted by 2 more exercises simultaneously to strengthen the hip abductor. One is lateral leg raise, in brief, the patients lie down on bed on the unaffected side, with the resistance band positioned around the distal thigh of the affected limb. Later, they raise the above lower limbs upwards for about 30 degrees, stay for 5–10 s, and slowly lay down. The other exercise is pelvic lift training, specifically, patients stand single-leg off the side at a 10-cm step. Later, they begin with the other limb that is lower than the step level, and contract the stance-limb hip abductor to raise the free leg to the step level while keeping the stance knee extended.

Patients will be required to carry out each exercise for 10 repeats at home as a set, for 3 sets each time and twice a day for 6 weeks altogether. Qualified physiotherapists who have attained the physical therapist’s certification with over 5 years of experience will be trained beforehand, so as to instruct and guide patients in carrying out these exercises. All exercises are standardized; however, the resistance for each movement should be decided according to the practical situation of each patient. As a result, all participants will have to visit the physiotherapists in the Affiliated Hospital of Southwest Medical University once a week for 6 weeks, so as to learn the exercises and adjust the training intensity. Typically, progression to a greater resistance level is possible when participants can perform 20 repeats of a given exercise without fatigue. Moreover, graded resistance elastic bands will be supplied to all participants who are also given a booklet with detailed description and pictures of the exercises, as well as a form on which they can record their completion everyday. Only those who have completed 80% of the target can be considered eligible and enrolled in the final statistics. Finally, all follow-up assessments will be assessed by a blinded physiotherapist.

### Outcomes

Participants will be assessed by other physiotherapists blinded to the group allocation at different time points based on different assessments shown in Table 1. Additionally, baseline age, gender, symptoms, disease severity (K-L grade), duration, previous treatment and medication will be recorded using a questionnaire (in week 0). The primary outcome is the overall pain assessed using VAS in the 6th week, while the secondary outcomes include the overall function measured by the Western Ontario and McMaster Universities Osteoarthritis Index (WOMAC), as well as the quality of life assessed by MOS Item Short-form Health Survey (SF-36), and several functional tests.

**Table 1:** Schedules for follow-up assessments and date collection

Assessments	Baseline	Active strengthening at home						Follow-up	
	Week 0	1st week	2nd week	3rd week	4th week	5th week	6th week	8th week	12th week
VAS for pain	√	√	√	√	√	√	√	√	√
WOMAC	√						√	√	√
SF-36	√						√	√	√
FTSST	√						√	√	√
Step test	√						√	√	√
Stair ascent/descent task	√						√	√	√
Figure-of-8 Walk Test	√						√	√	√
Adverse effects		√	√	√	√	√	√	√	√

The overall pain intensity in the involved knee within the past 1 week will be self-measured by patients using the VAS, with terminal descriptors ranging from 0 (no pain) to 10 (maximal pain). Meanwhile, the overall function of patients will be assessed adopting WOMAC, a disease-specific scale with high reliability and validity [23]. WOMAC is comprised of 24 items assessing pain (scored 0–20), stiffness (scored 0–8) and physical functions (including personal care, walking, sitting, lifting, sleeping, standing, social life and travelling) (scored 0–68). Every item is rated on a scale of 0–5, with a higher score indicating a worse symptom. Moreover, the quality of life will be measured by SF-36 constituted by eight dimensions, including mobility, pain and discomfort, daily activities, depression and anxiety, and self-care [24].

Furthermore, the Five Times Sit to Stand Test (FTSST) is used as a simple objective test of lower limb function [25]. To be specific, participants sit on an armless chair with their backs resting against the chair; later, they will be asked to stand up straight and sit down without touching the chair back each time. The test is accomplished upon the fifth time of returning to sitting, and the time spent will be recorded as a result. Besides, the step test is also employed to measure the standing balance [26]. In brief, participants stand barefoot on the diseased leg facing a 15 cm step; subsequently, they step the opposite foot on and off the step as quickly as possible. The frequency completed within 15 s will be recorded as a result, and a higher score indicates a better standing balance function. At the same time, the stair ascent/descent task [27] is also adopted in the current study, in which all participants will be invited to climb up and down a line of stairs, with eight steps one time at their usual speed. The time spent will be recorded as a result, and a longer time suggests a poorer physical function. In the Figure-of-8 Walk Test, the time that participants take to walk a path around 2 cones that is placed 1.5 m apart will be recorded [28]. Patients will be asked to stand in the middle of the 2 cones initially before they walk in a continuous figure-of-8 pattern around the cones. Finally, they will be asked to stop at the location where they begin, and the time spent will be recorded.

Moreover, the adverse effects, medication and other therapies in each participant over the course of trial will be recorded by themselves.

### Sample size

The sample size is calculated with reference to relevant previous studies. Notably, a change in VAS of knee pain is estimated to be 2.0 between two groups after the strengthening program, which suggests clinically important change. Meanwhile, the standard deviation (SD) between the experiment and the control groups is estimated to be 2.5 based on relevant previous publications [20]. Moreover, the sample size required for each group is about 33 when a two-tailed test with the power of 90% and the significance level of 5% (α error) is applied. Eventually, the total sample size is calculated to be 80 after taking into consideration a 1:1 allocation ratio and a dropout rate of 20%.

### Statistical analysis

Statistical analyses will be performed following the per-protocol (PP) analysis and intention-to-treat (ITT) analysis. Typically, PP analysis would be conducted if no participants have dropped out of the trial for any reason. In addition, the rule of last-observation-carried-forward will be applied for the missing data when performing ITT analysis. Additionally, baseline data will be analyzed and adjusted using the covariance analysis if no statistically significant differences between two groups and a possibility of covariance of baseline data are found. Categorical data, such as gender or those expressed as frequencies (%) will be analyzed using chi-square test or Fisher’s exact test. Non-parametric statistical tests will be applied for data not conforming to normal distribution. In addition, statistically significant differences in intra-group and intergroup data will be analyzed by repeated measure analysis of variance. All statistical analyses will be performed using the SPSS 19.0 statistical software by a statistician blinded to the allocation. The significance level is set at 5%.

### Safety

All exercises adopted in this study are very safe and harmless, especially under the guidance of physiotherapists. However, all expected or unexpected adverse events deriving from this study, such as muscle strain or ache, will be recorded and monitored. Moreover, patients subjected to harm from this study will receive free treatment, and any differences in safety between the two groups will be reported by researchers as a part of the results.

## Discussion

A single-blind randomized controlled trial is designed in the current study to examine whether quadriceps combined with hip abductor strengthening is superior to quadriceps strengthening alone in alleviating pain and improving physical function as well as the quality of life of KOA patients.

Researchers and clinicians have attached growing importance to lower limb muscle strengthening, which can be attributed to its effectiveness in reducing knee pain and improving physical function. Moreover, such a phenomenon is also linked to the economical efficiency of lower limb muscle strengthening and the possibility to be widely promoted among patients, especially those in communities. The lower extremities have formed a whole kinematic chain, which makes it impossible for the hip, knee or ankle joints to work completely independent from each other. Instead, they may affect each other.

As is noted in some studies, strength of the hip muscles, especially the hip abductor, may be altered in KOA patients. For instance, a research finds that the isokinetic strength of hip abductor in KOA group is remarkably lower than that in control group [19]. Another study shows that the explosive force and endurance, as well as the isokinetic strength of the hip abductor in KOA group are lower than those in control group [17]. Similarly, it is also found that the hip abductor in KOA patients is reduced by 24% compared with that in normal controls. Meanwhile, the hip abductor muscle strength is negatively correlated with the severity on KOA imaging findings, indicating that the more severe KOA imaging findings are related to the weaker hip abductor strength [16]. Not only the isokinetic strength, but also the isometric strength of hip abductor in KOA patients are smaller than normal, but the difference between two groups is not statistically significant [29]. Typically, the hip abductor plays an important role in stabilizing the trunk and pelvis in gait cycle. Therefore, weakness of hip muscle may lead to changes in the position of body center, resulting in contralateral movement of the pelvis or lateral leaning of the trunk over the standing limb. This would thus increase the magnitude of knee adduction moment, which is an indicator of disease progression. Consequently, it seems that hip abductor appears to have certain impact on knee joint load, which may also play a potential role in the symptoms and progression of disease.

Importantly, limited studies have pointed out that, hip abductor strengthening exercises may be helpful to improve the physical function and alleviate pain in KOA patients.

It was observed that the muscle strength of hip abductor is markedly enhanced, while the WOMAC-pain of knee and FTSST score are distinctly different before and after an 8-week home strengthening program for the hip abductor in 40 KOA patients [19]. Nonetheless, in that study, the patricians in control group are normal persons without KOA. Moreover, a study aiming at KOA patients discovers that, compared with the control group, hip muscle strength, WOMAC- pain, scores of the step test and the stair ascent/descent task are markedly improved in the training group receiving a 12-week hip muscle strength training [20]. It is also found that hip abductor training combined with the quadriceps femoris and hamstring muscle strength training can decrease the WOMAC-pain score in KOA patients by 78% [21] . However, the sample size is as small as 6, and no control group is set up [21]. Moreover, for patients undergoing total knee replacement, the muscle strength of hip abductor is also related to the functional activities (the Figure-of-8 Walk Test, the stair ascent/descent task, and FTSST) in addition to the quadriceps femoris [28]. Those studies have indicated that hip abductor muscle strength training may be beneficial to KOA patients. Therefore, we hypothesize that quadriceps combined with hip abductor strengthening may be superior to quadriceps strengthening alone for KOA patients, and the current study is thereby designed on this account.

Furthermore, the clinical changes in KOA-associated symptoms resulted from hip abductor strengthening intervention will be confirmed using the self-reported pain, as well as subjective measures of function and quality of life. Typically, the common and most widely used tools, including VAS, WOMAC and SF-36, are employed in the current study to measure the changes in symptoms and quality of life in KOA patients.

Besides, four simple tests that can be easily completed in the clinical hospital are selected as the objective measures of function, which have focused on different aspects, including the balance, walking ability, and flexibility. Therefore, the effects of hip abductor strengthening on the improvements of symptoms and quality of life in KOA patients can be evaluated in this study.

## Abbreviations

FTSST: Five times sit-to-stand test
KOA: Knee osteoarthritis
SF-36: MOS item short form health survey
VAS: Visual analogue scale
WOMAC: Western Ontario and McMaster Universities Osteoarthritis Index
